# Editorial: Interfacial Water: A Physical Chemistry Perspective

**DOI:** 10.3389/fchem.2020.00760

**Published:** 2020-09-02

**Authors:** Motomu Tanaka, Hideki Seto

**Affiliations:** ^1^Institute of Physical Chemistry, Heidelberg University, Heidelberg, Germany; ^2^Institute for Advanced Study, Kyoto University, Kyoto, Japan; ^3^High Energy Accelerator Research Organization, Tsukuba, Japan

**Keywords:** interfacial water, hydration, aquatic functional materials, surface and interface, hydrogen bonding, liquid-liquid phase separation

Earth is called a “water planet,” as it is the only planet possessing massive quantities of liquid water on its surface. Water is essential to sustain our life, as 60–65 wt% of a human body consists of water. Because of the increasing demand for more efficient use and management of water, the United Nations defines “Clean Water and Sanitation” as one of the Sustainable Development Goals (SDG 6).

Liquid water is one of the most ubiquitous materials on earth and is closely associated with life. Its anomalous behavior is recognized as the origin of a variety of phenomena in chemistry, biology, and geosciences and the underlying aspects of liquid water have been investigated from the viewpoint of physics. However, for reasons that include a lack of full understanding of liquid water (despite the accumulation of a vast body of experimental and theoretical data), the role of water at interfaces has yet to be fully elucidated. For example, ample evidence suggests that water molecules near the interface take various transient structures depending on the interacting surface, while the presence of water molecules significantly modifies the physical properties of surfaces.

This special issue of *Frontiers in Chemistry—Physical Chemistry and Chemical Physics* aims to shed light on interfacial water from different angles.

Tanaka (article 165) provided a comprehensive review on how the interfacial “forces” are counter-balanced and define the structure and mechanical properties of soft matter and biological matter in aquatic environments. Following the classical concept of *disjoining pressure* described by Derjaguin, this article exemplifies several surface sensitive techniques, such as high energy X-ray reflectivity and specular/off-specular neutron scattering, that enables the quantification of molecular-level structures and significance of interactions in aquatic environments. The perspective article by Cao et al. (article 626) further extends the topic to “looking at water near the solid/liquid interface.” They nicely summarize recent progress in non-contact atomic force microscopy and simulations enable to visualize hydrogen bonding network and weakly bonded water clusters. They conclude that the continuous improvement of AFM imaging and AFM modeling will bring about more comprehensive understanding of the structural, mechanical, dynamic, and functional heterogeneity of intricate interfacial water systems.

In this Research Topic, two papers deal with physical properties of water molecules interacting with biological membranes. Hydration of biological membranes is a biologically relevant issue, as many of key biochemical reactions are confined to the close proximity of membrane surfaces. Yamada and Seto's minireview (article 8) provides a compact summary of the application of the quasi-elastic neutron scattering (QENS) technique on hydration between lipid membranes. Such techniques, quantifying the diffusive dynamics in explicit geometry, will help material scientists to unravel the dynamics of water confined in nanochannels, such as polymer-based electrolytes for low-temperature fuel cells. Meineke et al. (article 455) also used neutron scattering to investigate the incorporated water between lipid membranes focusing on ice formation depending on the cooling rate at cryo-temperatures (100–300 K) using a membrane diffractometer. They found that a highly viscous phase of water exists between 180 and 220 K in flash-cooled samples due to the outflow of water from the inter-membrane space. This result suggests that water within DMPC membranes is less hindered in its movements than in the purple membrane case.

The other two papers provide with unique colors to this Research Topic. In their article (article 731), Nakagawa and Oyama examined the physical properties of water in glycerol-water mixtures in order to clarify the molecular basis of water activity by means of calorimetry, vibrational spectroscopy, and incoherent quasi-elastic neutron scattering. They have shown that three regions can be distinguished in the sorption isotherm and the relation with the water clustering and network formation as well as the interaction of water with glycerol. Since a high water activity leads to the formation and breaking of hydrogen bonds resulting in fast food degradation, this result may help food industry to optimize the conditions for food preservation. In the other paper (article 788), Mikuchi et al. reported spatio-temporal patterns of oil droplets on the water surface, which is one of the simplest examples of pattern formation in an open, non-equilibrium state. Oil droplets containing anionic surfactants undergo periodic deformation when deposited on the surface of water containing cations. They found that the oscillation pattern appears to depend on the cation species in the water phase. The experiments using such simple model systems might be helpful to gain a general perspective on spontaneous motion and deformation at physico-chemical energies, which has been drawing attention in physical chemistry of active matter.

Yet, little is understood about the structure, dynamics, and mechanics of interfacial water. Recent developments in experimental techniques and modeling tools would further foster our understanding of the physical chemistry of interfacial water in a broader context.

## Author Contributions

All authors listed have made a substantial, direct and intellectual contribution to the work, and approved it for publication.

## Conflict of Interest

The authors declare that the research was conducted in the absence of any commercial or financial relationships that could be construed as a potential conflict of interest.

